# Vocational Rehabilitation and Length of Stay at Work after Work-Related Musculoskeletal Disorders: A Longitudinal Study in Brazil

**DOI:** 10.3390/ijerph20032334

**Published:** 2023-01-28

**Authors:** Cristiano Barreto de Miranda, João Silvestre Silva-Junior, Klauss Kleydmann Sabino Garcia, Flávia Nogueira e Ferreira de Sousa, Frida Marina Fischer

**Affiliations:** 1 Department of Environmental Health and Graduate Program in Public Health, School of Public Health, University of São Paulo (FSP-USP), São Paulo 01246-904, Brazil; 2 Department of Forensic Medicine, Bioethics, Occupational Medicine and Physical Medicine and Rehabilitation, University of São Palo Medicine School (FMUSP), São Paulo 01246-903, Brazil; 3 General Coordinator of Occupational Health, Ministry of Health, Graduate Program in Tropical Medicine, University of Brasília (UnB), Brasília 70910-900, Brazil

**Keywords:** vocational rehabilitation, return to work, work-related musculoskeletal disorders, occupational health

## Abstract

Vocational rehabilitation is an intervention to enhance the return to work and improve quality of life. The aim of this study was to evaluate sociodemographic and occupational factors associated with the length of stay at work among workers with work-related musculoskeletal disorders (WRMDs) who had undergone rehabilitation through the Brazilian public social security system. This was a longitudinal study among 680 workers with histories of disability due to WRMDs who returned to the formal job market after vocational rehabilitation between 2014 and 2018. Survival analysis was performed to identify the factors influencing permanence in work. Job dismissal occurred for 29.26% of the workers. The average duration of employment after returning to the formal job position was 56 months. The following factors were associated with shorter length of employment: living in the southeastern region (HR: 2.78; 95% CI 1.12–6.91) or southern region (HR: 2.68; 95% CI 1.04–6.90) of Brazil; working in transportation, storage or postal services (HR: 2.57; 95% CI 1.07–6.17); or working in financial activities, insurance or related services (HR: 2.70; 95% CI 1.05–6.89). These findings may contribute to the discussion about prevention of disability and interventions to ensure health care for workers with WRMD disabilities who undergo rehabilitation.

## 1. Introduction

Work-related musculoskeletal disorder (WRMD) is a term for injuries caused by repetitive tasks, intense exertion, mechanical compression, vibrations or sustained postures in occupational settings [1,2]. In addition to biomechanical risks, WRMDs are associated with organizational and psychosocial factors present in the work environment [3,4,5]. They are characterized by occurrences of several symptoms (which may or may not be concomitant), such as chronic pain, paresthesia, muscle discomfort and muscle fatigue, which are manifested mainly in the neck, shoulder girdle, upper limbs, lumbar spine region and knees [1,5].

WRMDs are the most significant cause of work-related illness in many countries. They negatively affect work capacity and quality of life, and are one of the main reasons for work disability [1,3]. In Brazil, data from the Information System on Notifiable Diseases (SINAN) of the Ministry of Health indicate that 104,665 cases of WRMD were reported between 2006 and 2021 [6]. It should be noted that, in Brazil, WRMDs are notifiable diseases that are monitored through a sentinel surveillance strategy, composed of healthcare units that identify, investigate and report WRMD cases.

Early diagnosis of WRMDs provides better results regarding the health of workers thus affected, especially when preventive treatment is implemented within the environment and work processes. On the other hand, non-preventive intervention and late diagnosis have been correlated with higher degrees of work disability and difficulties in returning to work [7,8].

Studies have shown that sociodemographic factors (gender, age and education) and occupational conditions (reduction in working hours and support from colleagues and bosses) can have an influence as facilitators or barriers to the effectiveness of returning to work after being away from work due to WRMDs [9,10,11].

When the disabling sickness evolves into a permanent condition and prevents workers from performing their usual functions, the Brazilian National Social Security Institute (INSS) may indicate placement in the vocational rehabilitation program. This public service offers educational assistance and professional adaptation (or readaptation) for insured individuals. During this rehabilitation, the disability aid is maintained until these individuals are considered to have achieved rehabilitation, such that they are able to perform an activity that ensures their subsistence. Otherwise, they are granted pensionable retirement on the grounds of permanent disability from which rehabilitation is impossible [12].

A previous study showed that the average cumulative incidence of rehabilitation was 57.4 per 1000 admissions to the Brazilian social security vocational rehabilitation program from 2007 to 2016 [13]. Although monitoring of rehabilitated workers who returned to work is recommended by social security, this practice is limited among vocational rehabilitation services, and information on rehabilitated workers reintegrated into work remains poor.

The main goal of the Brazilian vocational rehabilitation program is to enable workers with disabilities to qualify for a new work activity. Therefore, studies that investigate occupational factors such as the occupations and economic activity sectors that allow greater possibilities for reentry and permanence in work are fundamental for guiding actions that promote a sustained return to work in a highly competitive labor market. Knowledge of the occupations and economic activity sectors into which rehabilitated workers are placed while developing their work activity can help in understanding the occupational risks to which these workers are exposed. This can provide support for effective intervention strategies in the work environment that promote and protect the health of these workers.

In addition, there are few studies on returning to and remaining in work after vocational rehabilitation in Brazil. Knowledge of the factors associated with the length of stay at work after vocational rehabilitation can help identify workers who are more vulnerable to unemployment and support construction of strategies to facilitate a sustained return to work. 

Therefore, the aim of this study was to evaluate sociodemographic and occupational factors associated with the length of stay at work among workers with disabilities due to WRMDs who had undergone rehabilitation through the Brazilian social security system.

## 2. Materials and Methods

### 2.1. Study Design and Population

This was a longitudinal study on workers with disabilities due to WRMDs who had undergone rehabilitation through the Brazilian Social Security system and who returned to the formal employment market between 2014 and 2018. The data referred to workers over 18 years old with an employment contract that was not time limited. Workers who had undergone rehabilitation and were holding more than one job, and workers with temporary employment were excluded from the study.

### 2.2. Data Sources

The 2018 Annual Social Information Report (RAIS) database, which contains retrospective information referring to previous years, was the primary data source. RAIS has nationwide coverage and provides information on formal employment relationships, with mandatory annual registration for public and private companies. RAIS is one of Brazil’s main population databases for workers in the formal employment market [14]. RAIS does not provide data on diagnoses of work-related illnesses.

The Information System for Work Accident Communications (SISCAT) database records work-related accidents and illnesses notified by private companies that contribute to the degree of incidence of labor disability resulting from environmental risks at work [15]. This database was used to identify diagnoses of workers with WRMDs over the period from 2014 to 2018.

The RAIS and SISCAT databases were linked together using the method described by Garcia et al. (2022) [16]. This linkage was performed and provided by the General Coordination Office for Workers’ Health of the Ministry of Health (CGSAT/MS) anonymously and without duplication, specifically for our study. Thus, we did not have access to any sensitive data on workers or companies.

Database linkage is a technique that enables correlations between different information sources in a single record [16]. A deterministic approach was used in this study: this identified pairs of completely concordant records through a standard identifier variable common to the different databases used [16]. The PIS (Social Integration Program) number was the key variable for making the linkage since it formed a unique identifier in the two databases studied.

Workers who had undergone rehabilitation through Social Security were identified by analyzing the variable “type of disability” in the RAIS database. This indicated whether a given worker currently presented a disability but was not participating in rehabilitation programs or whether this worker had completed the rehabilitation. The diagnosis of the WRMD was identified through the International Classification of Diseases version 10 (ICD-10), in chapter XIII, as codes M00 to M99 (from the SISCAT database). This information is not present in the RAIS database.

### 2.3. Study Variables

The dependent variable was “length of stay at work after returning”, which was the total number of months that elapsed between the worker’s date of admission to a job and the date of dismissal. The study period was 60 months from the date on which each rehabilitated worker returned to employment.

The following sociodemographic variables were analyzed: sex, age group, race/skin color, number of years of schooling and Brazilian macroregion of residence/work.

The following occupational variables were studied: average monthly salary range, company size, weekly working hours, occupation (classified based the Brazilian Classification of Occupations (CBO-2002)) and economic activity (classified based on the National Classification of Economic Activities (CNAE-2.0)). The CBO is the standardizing document for the recognition, naming and codification of the titles and contents of the occupations in the Brazilian labor market. 

The CBO is structured in 10 major groups (one digit), 47 main subgroups (two digits), 193 subgroups (three digits), 596 occupation families (four digits) and 2666 occupations (six digits). For better data visualization, it was opted here to choose the occupation designated by the 10 major groups of the CBO. The CNAE is structured in 21 sections (one letter), 87 divisions (two digits), 286 groups (three digits), 673 classes (four digits) and 1302 subclasses (seven digits). For better data visualization, it was opted here to choose the economic activities designated by the 21 sections of the CNAE. 

### 2.4. Statistical Analysis

A descriptive analysis on the distribution of the absolute and relative frequencies of all the variables studied was conducted. A survival analysis, in which cases of workers who were fired were considered to be “failures”, was also conducted. The number of months between the date of job admission and the date of dismissal was recorded. Right censoring was undertaken the end of the follow-up, i.e., 60 months after the return to work, and in cases in which the dismissal occurred due to death or retirement.

Survival curves were constructed for the covariates using the Kaplan–Meier method [17]. To test whether the duration of employment differed between the categories of a given risk or protection factor (for example, between males and females), the log-rank test was used [18].

The Cox proportional hazards model was applied to the study variables in order to estimate the risk of dismissal from the job. Univariate analysis on each covariate was performed. In constructing the multiple model, variables in the univariate analysis that presented a significance level of *p* < 0.20 were included. The stepwise forward method was used, starting from the saturated model, until it was identified which model would explain most of the variance. The likelihood ratio of the proposed model in relation to the saturated model was used to assess the fit of the model (deviance analysis). For the final model, variables whose *p*-value was <0.05 were taken to be statistically significant. To evaluate the premise of the final model regarding the proportionality of risk, the Schoenfeld test was performed [19].

The hazard ratio expressed the association between the study variables and the outcome, with their respective 95% confidence intervals. A hazard ratio (HR) greater than one (HR > 1) denoted a greater risk of being dismissed from employment during the follow-up. HR < 1 denoted a lower risk of being dismissed from work during the study period [19].

### 2.5. Ethical Considerations

The Research Ethics Committee of the School of Public Health of the University of São Paulo (CAAE: 04267218.0.0000.5421, opinion number: 5113575) approved this study.

## 3. Results

Most of the 680 workers who underwent rehabilitation and then returned to the formal employment market between 2014 and 2018 were male (62.35%), had white skin color (52.79%), had up to 11 years of schooling (62.21%) and were living in the southeastern region of the country (41.18%). The workers’ mean age was 44.68 years (SD = 8.30) and there was no statistical difference in the mean age between the sexes (*p* = 0.459) (Table 1).

Most of the rehabilitated workers (46.47%) had an average monthly remuneration in the range of 2.01 to 4 times the minimum wage (USD 400–800), were employed in a large company (44.41%) and worked for more than 40 h per week (80.29%). The most frequently observed occupational group was administrative service workers (70.44%) and the most frequently observed economic activity sector was transportation, storage and postal services (52.35%) (Table 2).

The most frequently observed musculoskeletal diagnosis was shoulder injury. This, together with synovitis, tenosynovitis and back pain, represented the majority (63.1%) of all musculoskeletal disorders (Table 3).

**Figure 1:**
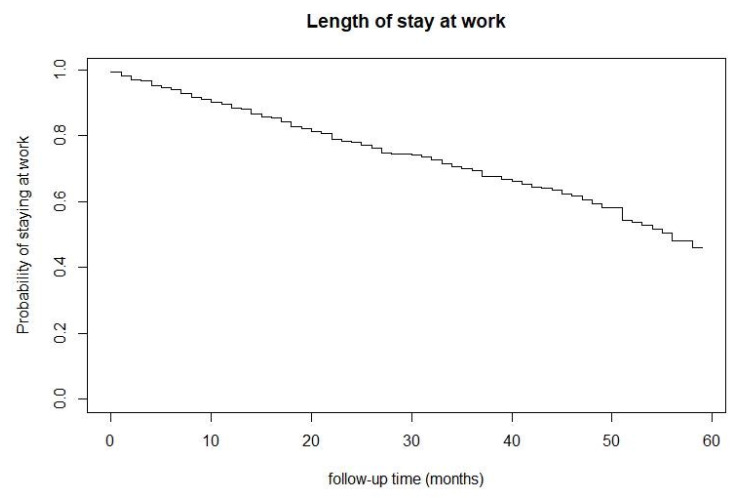
Probability of staying at work. Source: RAIS, 2018; SISCAT, 2016–2018.

The median time spent in work was 56 months (SE: 0.04) (Figure 1).

Table 4 presents the results from univariate and multiple analyses on the associations between the length of time spent in work and the covariates studied. The variables selected for the multiple models were sex, race/skin color, number of years of schooling, geographical macroregion, company size, occupational group and economic activity group.

The final model was fitted according to the workers’ sex, and the probability of agreement estimated through the model had a discriminatory or predictive value of 65.3%.

Thus, our findings showed that workers with disabilities due to WRMDs who returned to work after vocational rehabilitation, and who were living in the southeastern region (HR: 2.78; 95% CI 1.12–6.91) and southern region (HR: 2.68; 95% CI 1.04–6.90) were at higher risk of dismissal from work than workers in the northern region. Likewise, rehabilitated workers in the economic activity sectors of transportation, storage and postal services (HR: 2.57; 95% CI 1.07–6.17) or financial activities, insurance and related services (HR: 2.70; 95% CI 1.05–6.89) were at higher risk of dismissal than those in the manufacturing sector (Table 4).

On the other hand, rehabilitated workers employed in large companies were 60% more likely to remain in work than those employed in microenterprises. Being a rehabilitated worker in the occupational group of science and arts professionals also increased the chances of remaining employed for a longer time by 12%, compared with the occupational group of managers (Table 4).

## 4. Discussion

This study showed that seven out of ten workers who underwent rehabilitation due to WRMD disabilities between 2014 and 2018 remained employed. The results also showed that workers in the southeastern and southern regions who worked within the economic activity sectors of transportation, storage and postal services, or financial activities, insurance and related services, had shorter periods of formal employment after returning to work.

It is not possible to broadly compare our findings with those from other populations because of differences between countries’ disability and return-to-work policies, along with methodological differences between studies. For example, a study comparing sustained return to work after two years of sick leave due to low back pain across six countries found a considerable difference in job retention, ranging from 22% among workers in a German cohort to 62% among workers in a Dutch cohort. Among the workers in the other four countries studied, the following results regarding permanence in employment were found: 31% in Denmark, 39% in Sweden, 49% in the USA and 49% in Israel [20].

In Brazil, rehabilitated workers who received social security benefits characterized as due to work-related sickness are entitled to job security for at least one year after the benefit ceases, according to social security legislation [12]. In addition, companies with 100 or more workers are legally required to fill 2% to 5% of their staff positions with rehabilitated professionals or people with disabilities [12].

In principle, the abovementioned legal measures favor permanence of rehabilitated employees in large companies, as seen in our results, which showed that the probability of remaining in the job in the first year after returning to work was greater than 80%. However, after this period, we observed that there was a constant decrease in the probability of remaining in work over the remainder of the months studied. This finding corroborates the results from a study by Vacaro and Pedroso (2011), which showed that there was a higher unemployment rate among workers who had undergone rehabilitation through the Brazilian social security vocational rehabilitation program one year after their return to work [21].

Sociodemographic characteristics such as sex, age group, race/skin color, income and number of years of schooling did not influence the sustainability of the return to work. Regarding sex, our findings do not corroborate previous studies that found that females were less likely to return to work [10,22]. However, when the duration of the sustained return to work was investigated, the influence of sex did not seem to be significant. A study carried out among workers with disabilities due to musculoskeletal disorders and mental disorders who were followed up for two years did not find any significant difference between men and women [23].

On the other hand, there is strong evidence that older individuals are less likely to return to work and stay at work [22,23,24]. Regarding the variables of number of years of schooling, race/skin color and income, the results available from previous studies were inconclusive, and divergences remain regarding the impact of these characteristics on reintegration into work [10,25,26].

Our findings also showed that living in the southeastern and southern regions of Brazil had a negative influence on staying at work. Although these regions have the best socioeconomic indicators in Brazil [27], the national economic crisis that began in 2015 has had a negative impact on this country’s employment rates, such that the rates of unemployment and underutilization of the labor force in the southeastern and southern regions are now higher than in other Brazilian regions [28]. This context of a crisis in the labor market, with greater impact in these regions, may explain the results found in our study. Workers with work disabilities could possibly have greater difficulty in competing for employment, in terms of access and maintenance in the job, compared with others who are considered healthy. A previous study in Italy showed that almost a quarter of workers with work disabilities were fired after returning to work, within a scenario of neoliberal measures to make the labor market more flexible [29]. 

It is essential to point out that in the case of workers with a history of work disability, as in cases of WRMD, returning to work does not imply total pain control. The goal is for them to reach a state in which stability of the clinical condition is achieved, which thus favors the return to work [30].

However, in a context of intensification and precariousness of work, the chances of staying in the job are almost nil. The pace of work and the various abusive pressures tend to worsen the clinical picture of the illness, thereby further reducing the individual’s ability to cope with the work [30]. Although the degree of disability among the rehabilitated patients was not evaluated in this study due to unavailability of this information in the databases investigated, a four-year cohort study conducted among nursing workers showed that nurses with impaired work ability were at higher risk of dismissal [31].

Regarding the relationship between the length of stay at work and the size of the company, it was found that rehabilitated workers in large companies stayed in the job for longer periods. This can be partly explained by the legal obligation mentioned above, in which large companies must allocate a percentage of vacancies to rehabilitated workers. Another explanation is that there is a greater possibility of finding compatible functions for rehabilitated workers in large companies.

Regarding occupational groups and the relationship with permanence in work, not many studies providing explanations about the occupations within which workers with disabilities due to musculoskeletal disorders would be at lower risk of remaining employed, in relation to economic activity sectors, could be found. However, evidence from previous studies has indicated that working in managerial and support activities that involve less interaction with the public presents a relationship with shorter times taken to return to work [32].

Our findings showed that rehabilitated workers engaged in “transportation, storage and postal service activities” and “financial activities, insurance and related services” remained employed for shorter times. This was possibly due to the characteristics of the activities involved in these areas, which particularly expose workers to a variety of occupational agents of a physical nature, such as noise and vibration; to ergonomic stressors at work, such as physical effort and load lifting; and to lower autonomy and control over productivity. Such jobs present higher stress levels and exacerbate these individuals’ degree of incapacity [33]. Studies assessing the environmental characteristics of work, along with aspects of the context and content of work tasks, are needed.

The strength of the present study is that it presents external validity, given that it encompassed nationwide data with a longitudinal design in determining the outcome. Few Brazilian studies have evaluated the permanence of individuals in work after completing the vocational rehabilitation provided through social security. One limitation of the present study was the impossibility of identifying the date of leaving the rehabilitation program. Thus, it was unknown whether this was the first return to work after vocational rehabilitation. Another limitation was that this study did not include workers in the informal job market or workers with disabilities due to musculoskeletal diseases that were unrelated to work.

## 5. Conclusions

The length of stay at work among workers with histories of disability due to WMSDs who were reintegrated into formal employment through rehabilitation provided through the Brazilian social security system was influenced mainly by occupational factors related to the size of the company, type of occupation and sector of economic activity. It was found that workers in large companies and in the occupational group of “science and arts professionals” remained employed for longer than workers in microenterprises and in the occupational group of “general managers”. Being a rehabilitated worker in the southeastern or southern region and carrying out economic activities of “transportation, storage and postal services” and “financial activities, insurance and related services” were factors that negatively influenced the length of stay in formal employment.

Although Brazilian legislation guarantees vacancies for rehabilitated workers, it is clear that this legislation needs to be more capable of broadly promoting these workers’ inclusion. Despite the impossibility of ascertaining the degree of disability of the study subjects, the functional limitations of these rehabilitated workers meant that they were barred from performing some tasks that required certain skills. They were consequently at a disadvantage with regard to entering and staying in a highly competitive labor market in which formal employment is increasingly fragile.

We hope that the findings from this study can contribute to discussion and elaboration of more effective vocational rehabilitation measures, with a view to changing the limited approach in which workers are already incapacitated with regard to remaining in work activity. Early interventions are often more successful in relation to preventing harmful work conditions and promoting a sustainable return to work.

## Figures and Tables

**Table 1:** Sociodemographic characteristics of workers with disabilities due to WRMDs who had undergone rehabilitation through Social Security and then returned to the formal employment market. Brazil, 2014–2018 (*n* = 680).

Features	*n* (%)
Sex	
Male	424 (62.35)
Female	256 (37.65)
Age (in years)	
Up to 40	216 (31.76)
More than 40	464 (68.24)
Race/skin color	
White	359 (52.79)
Nonwhite	321 (47.21)
Number of years of schooling	
Up to 11	423 (62.21)
Above 11	257 (37.79)
Geographical region	
North	29 (4.26)
Northeast	188 (27.65)
Southeast	280 (41.18)
South	138 (20.29)
Center-west	45 (6.62)

Source: RAIS, 2018; SISCAT, 2016–2018.

**Table 2:** Occupational characteristics of workers with disabilities due to WRMDs who had undergone rehabilitation through Social Security and then returned to the formal employment market. Brazil, 2014–2018 (*n* = 680).

Features	*n* (%)
Average monthly remuneration (times minimum wage)	
under 2	211 (31.03)
from 2 to 4	316 (46.47)
above 4	153 (22.50)
Company size	
Microenterprise	115 (16.91)
Small company	185 (27.21)
Medium company	78 (11.47)
Large company	302 (44.41)
Weekly working hours	
≤40	134 (19.71)
over 40	546 (80.29)
Occupation	
Administrative service workers	479 (70.44)
Workers within production of goods, industrial services, repair and maintenance	71 (10.44)
Midlevel technicians	41 (6.03)
Science and arts professionals	34 (5.00)
Service workers and sales workers in stores and markets	32 (4.71)
General managers	23 (3.38)
Economic activity	
Transportation, storage and postal services	356 (52.35)
Financial activities, insurance and related services	140 (20.59)
Processing industries	63 (9.26)
Other economic activities	40 (5.88)
Sale and repair of motor vehicles and motorcycles	39 (5.74)
Administrative activities and complementary services	22 (3.24)
Human health and social services	20 (2.94)

Source: RAIS, 2018; SISCAT, 2016–2018.

**Table 3:** Distribution of workers who underwent rehabilitation through Social Security and then returned to the formal employment market, according to their primary clinical diagnoses of disability due to musculoskeletal disorders. Brazil, 2014–2018 (*n* = 680).

Clinical Diagnosis According to ICD-10 Chapter XIII, Codes M00-M99	*n* (%)
M75—Shoulder injuries	154 (22.65)
M65—Synovitis and tenosynovitis	141 (20.74)
M54—Back pain	134 (19.71)
M51—Other intervertebral disc disorders	63 (9.26)
M69—Other joint disorders not elsewhere classified	29 (4.26)
M23—Internal disorders of the knees	27 (3.97)
M77—Other enthesopathy	24 (3.53)
M50—Cervical disc disorders	18 (2.65)
M79—Other soft tissue disorders not elsewhere classified	12 (1.76)
M16—Coxarthrosis	11 (1.62)
Other musculoskeletal diseases	67 (9.85)

Source: RAIS, 2018; SISCAT, 2016–2018.

**Table 4:** Univariate and multiple analyses (Cox regression) on the length of stay at work among workers who underwent rehabilitation through Social Security due to WRMD disabilities, who then returned to the formal employment market. Brazil, 2014–2018.

Features	Univariate		Multiple	
	HR	95% CI	HR	95% CI
Sex				
Female	1.00	-	1.00	-
Male	1.05	0.79–1.40	0.99	0.73–1.37
Age group				
Up to 40 years	1.00	-		
Over 40 years	0.98	0.73–1.32		
Race/skin color				
White	1.00	-		
Not White	0.82 ^b^	0.62–1.90		
Number of years of schooling				
Up to 11 years	1.00	-		
Over 11 years	1.47 ^a^	1.11–1.95		
Geographical region				
North	1.00	-	1.00	-
Northeast	1.68	0.67–4.20	2.29	0.90–5.80
Southeast	2.41 ^b^	0.98–5.94	2.78 ^a^	1.12–6.91
South	2.08	0.82–5.28	2.68 ^a^	1.04–6.90
Center-west	2.15	0.78–5.91	2.41	0.87–6.72
Average monthly salary				
Up to 2 times minimum wage	1.00	-		
From 2.01 to 4 times minimum wage	0.79	0.57–1.08		
Above 4 times minimum wage	0.87	0.60–1.28		
Company size				
Microenterprise	1.00	-	1.00	-
Small company	0.87	0.60–1.28	0.86	0.57–1.28
Medium company	0.82	0.51–1.30	0.84	0.52–1.35
Large company	0.41 ^a^	0.28–0.61	0.60 ^a^	0.38–0.92
Weekly working hours				
Up to 40 h	1.00	-		
Over 40 h	1.15	0.79–166		
Occupation				
General managers	1.00	-	1.00	-
Science and arts professionals	0.12 ^a^	0.02–0.48	0.12 ^a^	0.02–0.50
Mid-level technicians	0.69	0.32–1.48	1.07	0.49–2.36
Administrative service workers	0.56	0.30–1.03	0.61	0.30–1.23
Service workers and salespeople in stores and markets	0.23 ^a^	0.07–0.71	0.50	0.14–1.75
Workers within production of goods, industrial services, repair and maintenance	0.26 ^a^	0.11–0.58	0.69	0.25–1.94
Economic activity				
Processing industries	1.00	-	1.00	-
Sale and repair of motor vehicles and motorcycles	1.82	0.76–4.40	1.92	0.73–5.06
Transportation, storage and postal services	2.92 ^a^	1.50–5.60	2.57 ^a^	1.07–6.17
Financial activities, insurance and related services	3.47 ^a^	1.74–6.94	2.70 ^a^	1.05–6.89
Administrative activities and complementary services	1.17	0.37–3.75	1.30	0.37–4.53
Human health and social services	0.55	0.12–2.52	0.46	0.09–2.35
Other economic activities	1.31	0.50–3.45	1.28	0.46–3.56

Adjusted for sex; ^a^, *p* < 0.05; ^b^, *p* < 0.20; HR: hazard ratio. Source: RAIS, 2018; SISCAT, 2016–2018.

## Data Availability

The data are not publicly available. A deidentified dataset can be obtained by contacting the first author.
